# Supplementary material for: Cannabidiol Suppresses Angiogenesis and Stemness of Breast Cancer Cells by Downregulation of Hypoxia-Inducible Factors-1α
Source: Cancers (Basel). 2021 Nov 12;13(22):5667. doi: 10.3390/cancers13225667 (PMC8616476; doi:10.3390/cancers13225667)

Supplementary Figures :

Cannabidiol suppresses angiogenesis and stemness of breast cancer cells by downregulation of hypoxia-inducible factors -1α

Min Jee Job[1]†, Bu Gyeom Kimb[1]†, Hye Kyeong Yun[1], Soyeon Jeong[2], Bo Ram Kim[2], Jung Lim Kim[2], Seong Hye Park[1], Dae Yeong Kim[1], Sun Il Lee[3], Sang Cheul Oh[1,2], Dae-Hee Lee[4]* , and Woo Young Kim[3]*

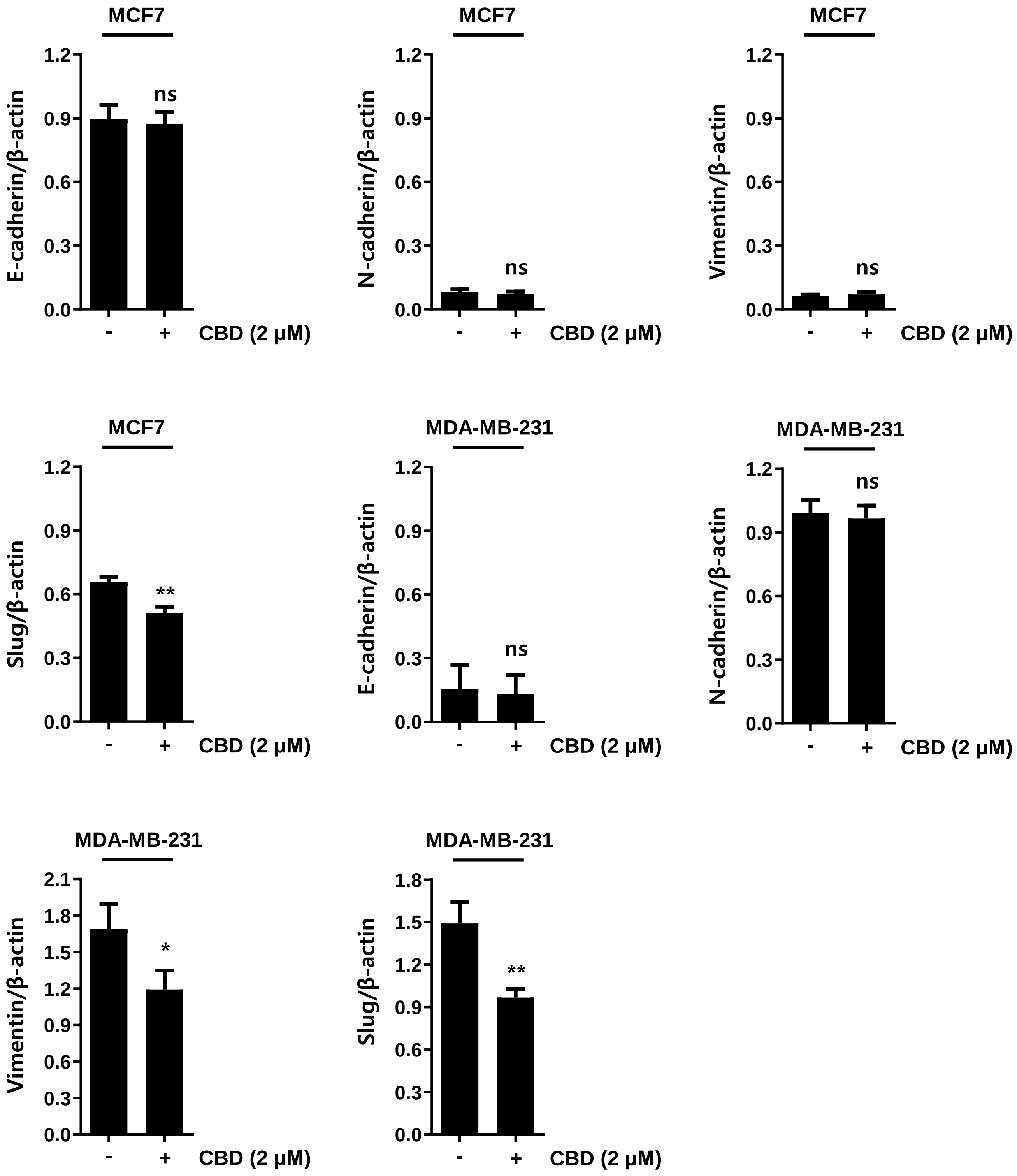

Figure S1. Quantifications and statistics of western blot assays relative to Figure 1B. Blots were quantified using ImageJ software and normalized with β-actin bands as a loading control. Statistical significance was defined as P values < 0.05 (*, **, and *** indicate $P < 0.05$, $P < 0.01$, and $P < 0.001$, respectively)

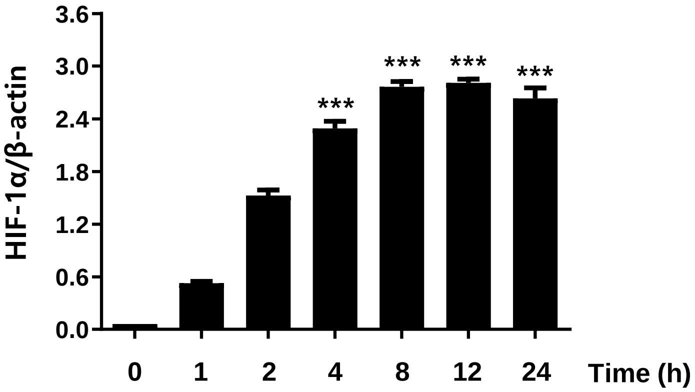

Figure S2. Quantifications and statistics of western blot assays relative to Figure 2A. Blots were quantified using ImageJ software and normalized with β-actin bands as a loading control. Statistical significance was defined as P values < 0.05 (*, **, and *** indicate P < 0.05, P < 0.01, and P < 0.001, respectively)

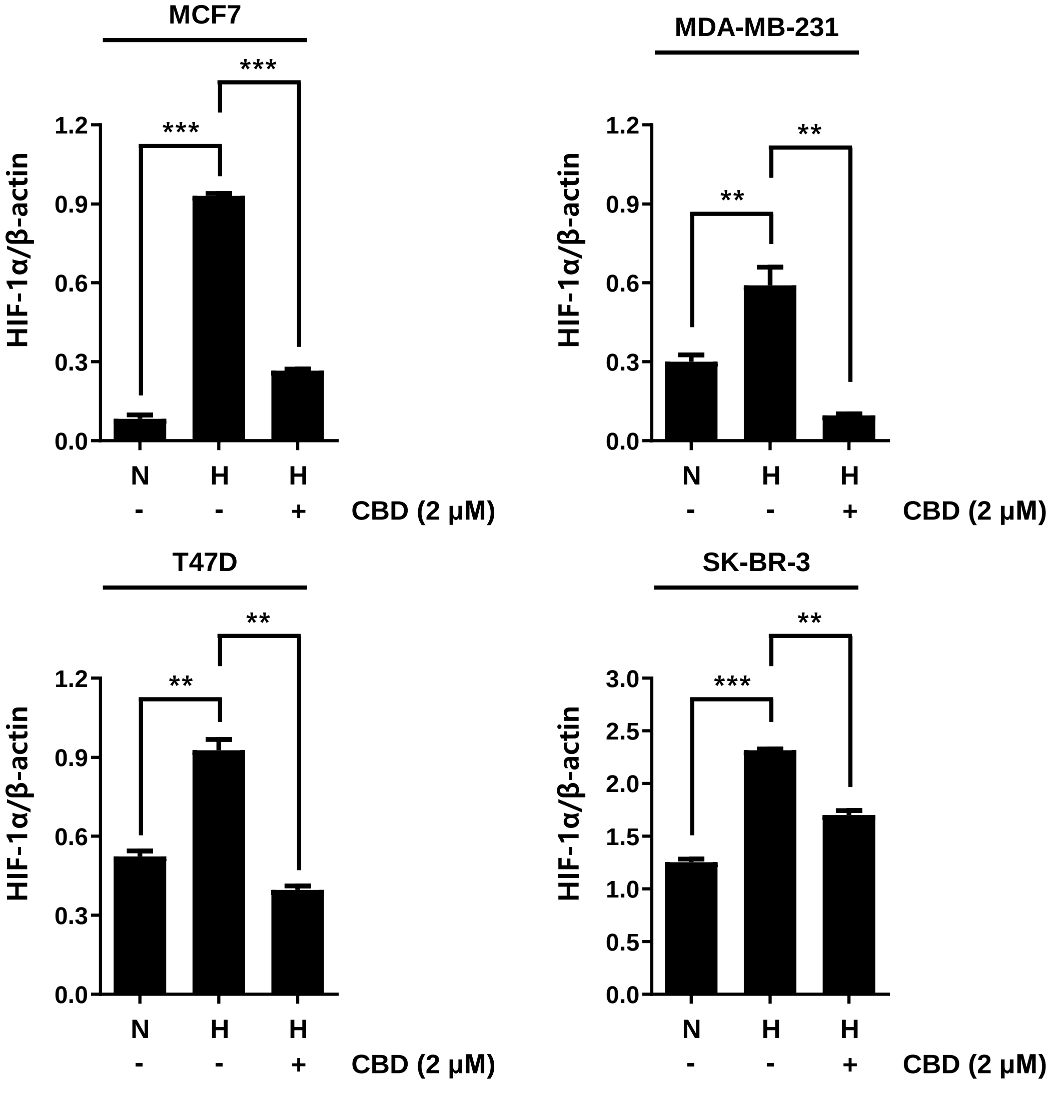

Figure S3. Quantifications and statistics of western blot assays relative to Figure 2B. Blots were quantified using ImageJ software and normalized with β-actin bands as a loading control. Statistical significance was defined as P values < 0.05 (*, **, and *** indicate P < 0.05, P < 0.01, and P < 0.001, respectively)

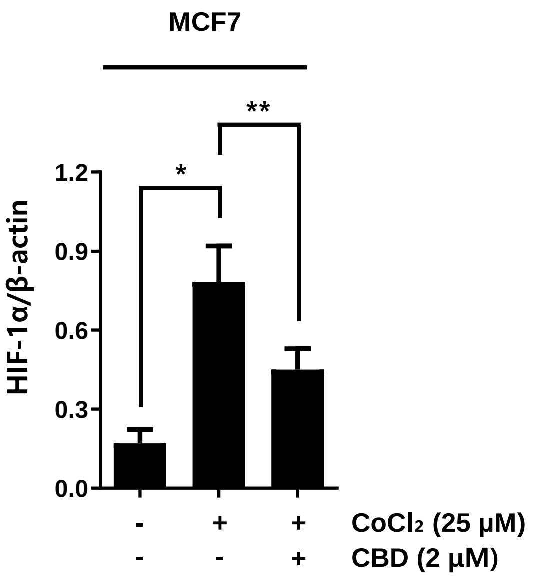

Figure S4. Quantifications and statistics of western blot assays relative to Figure 2C. Blots were quantified using ImageJ software and normalized with β-actin bands as a loading control. Statistical significance was defined as P values < 0.05 (*, **, and *** indicate P < 0.05, P < 0.01, and P < 0.001, respectively)

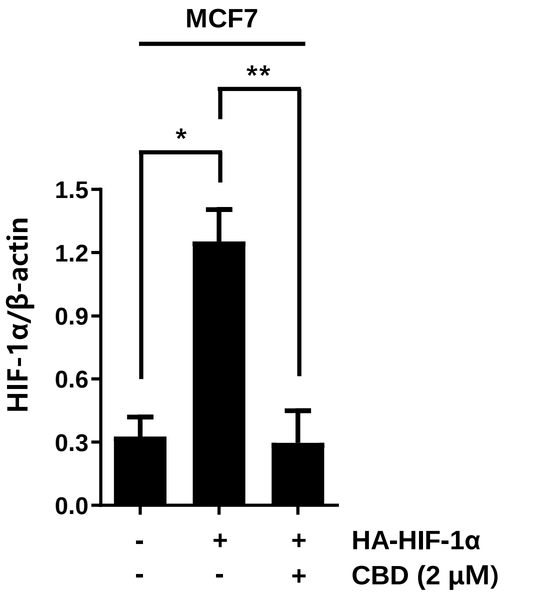

Figure S5. Quantifications and statistics of western blot assays relative to Figure 2D. Blots were quantified using ImageJ software and normalized with β-actin bands as a loading control. Statistical significance was defined as P values < 0.05 (*, **, and *** indicate P < 0.05, P < 0.01, and P < 0.001, respectively)

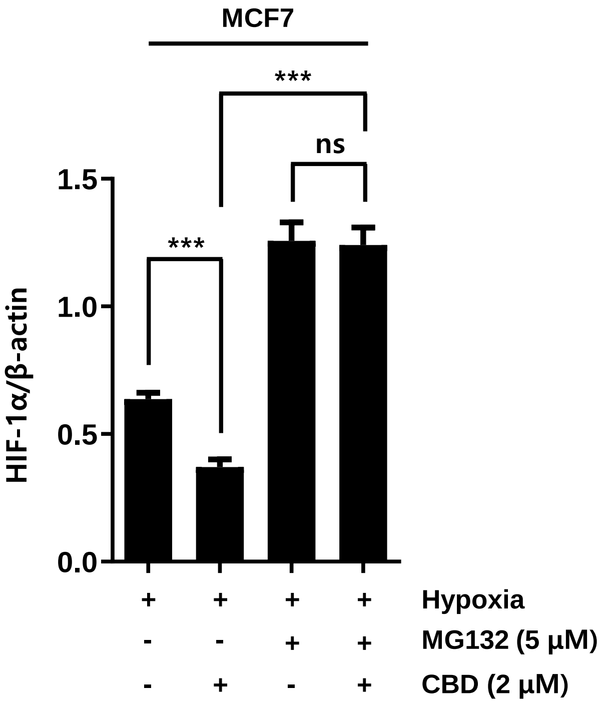

Figure S6. Quantifications and statistics of western blot assays relative to Figure 2G. Blots were quantified using ImageJ software and normalized with β-actin bands as a loading control. Statistical significance was defined as P values < 0.05 (*, **, and *** indicate P < 0.05, P < 0.01, and P < 0.001, respectively)

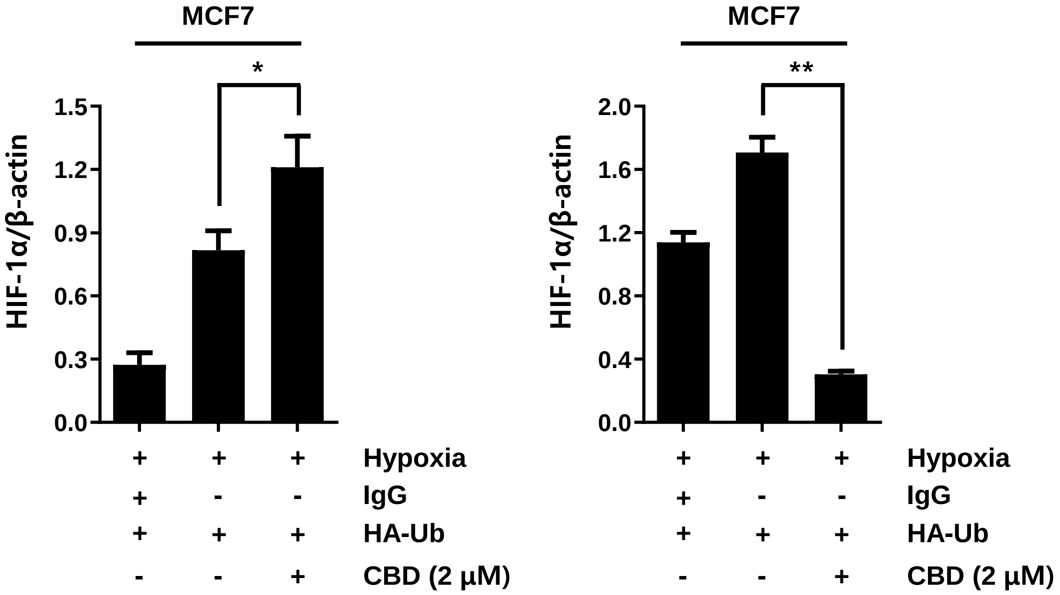

Figure S7. Quantifications and statistics of western blot assays relative to Figure 2H. Blots were quantified using ImageJ software and normalized with β-actin bands as a loading control. Statistical significance was defined as P values < 0.05 (*, **, and *** indicate P < 0.05, P < 0.01, and P < 0.001, respectively)

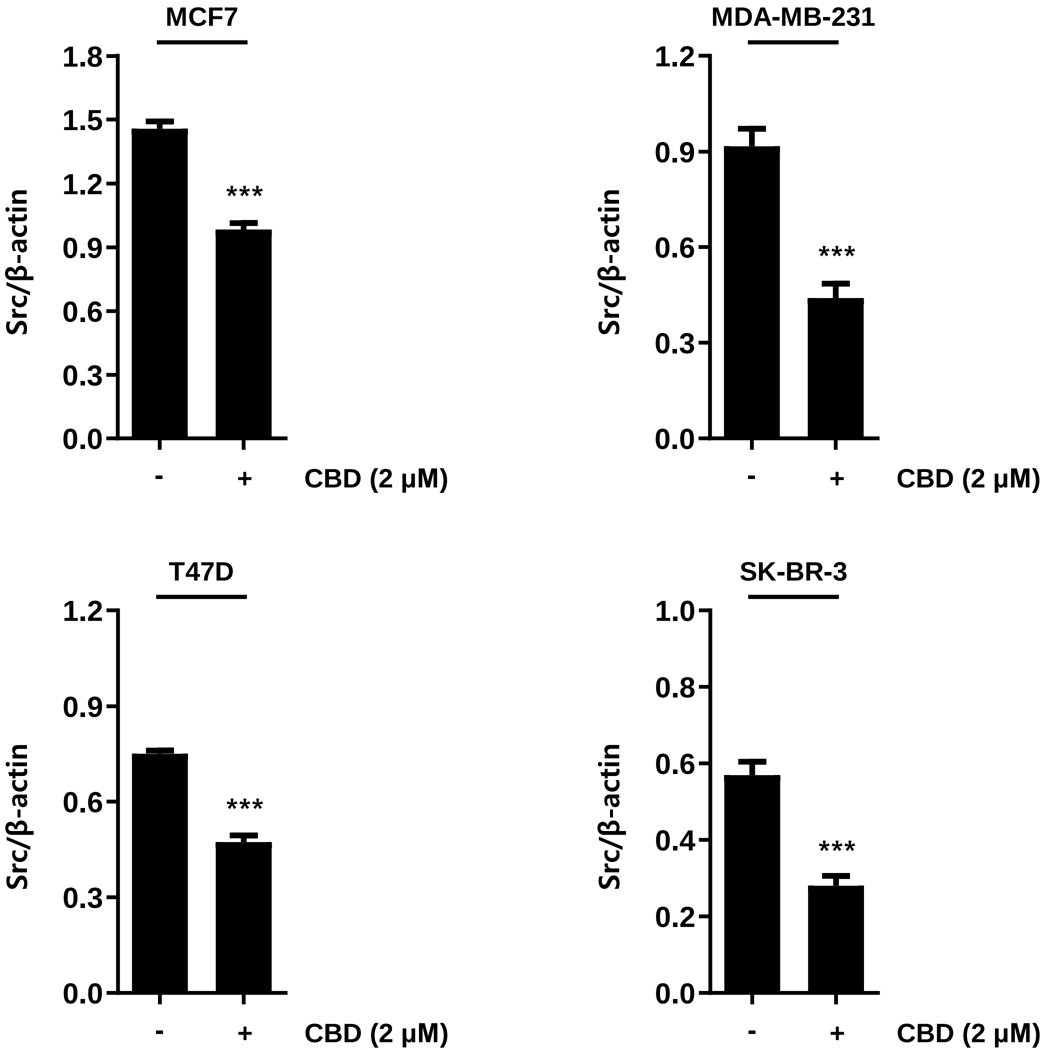

Figure S8. Quantifications and statistics of western blot assays relative to Figure 3A. Blots were quantified using ImageJ software and normalized with β-actin bands as a loading control. Statistical significance was defined as P values < 0.05 (*, **, and *** indicate $P < 0.05$, $P < 0.01$, and $P < 0.001$, respectively)

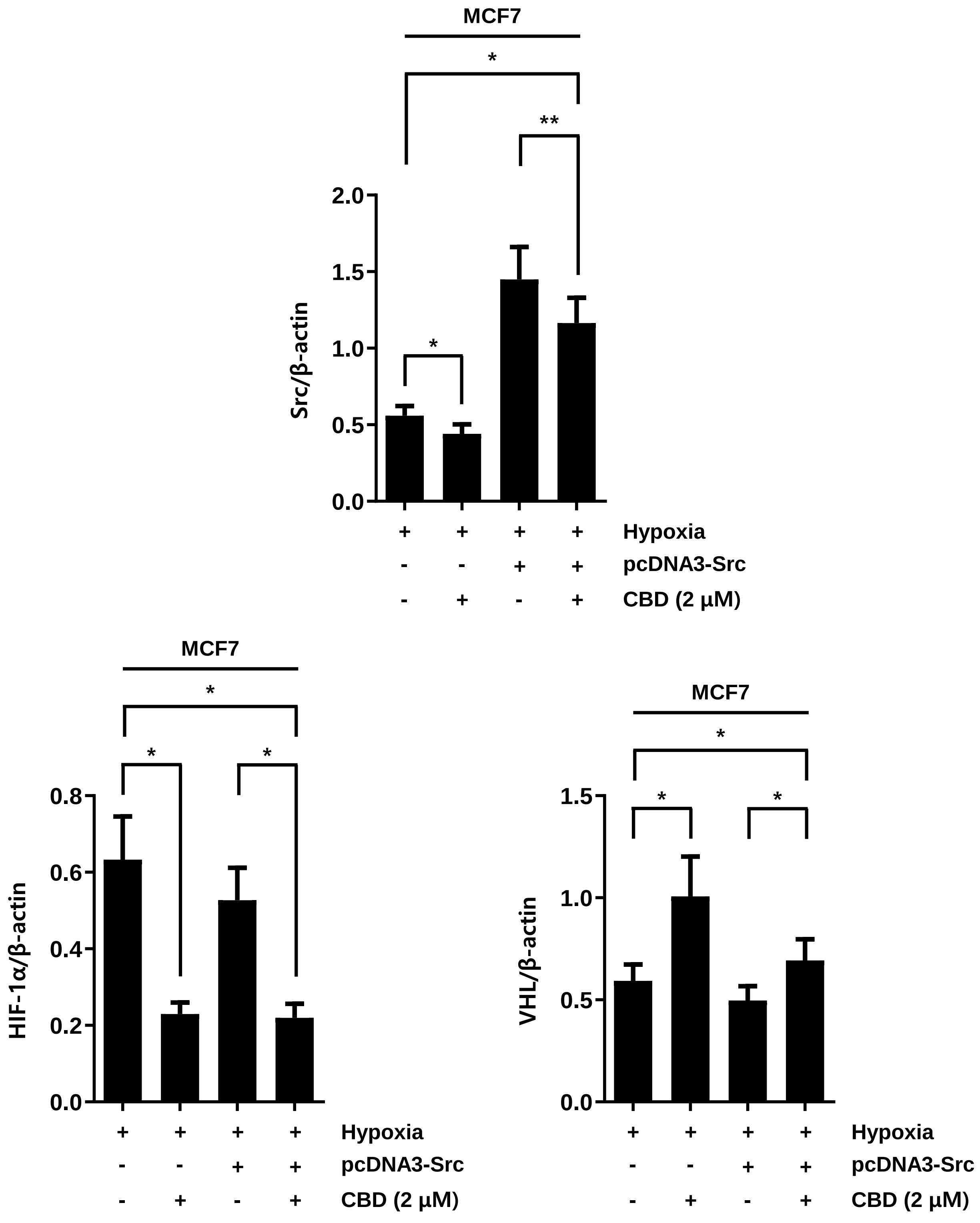

Figure S9. Quantifications and statistics of western blot assays relative to Figure 3C. Blots were quantified using ImageJ software and normalized with β-actin bands as a loading control. Statistical significance was defined as P values < 0.05 (*, **, and *** indicate P < 0.05, P < 0.01, and P < 0.001, respectively)

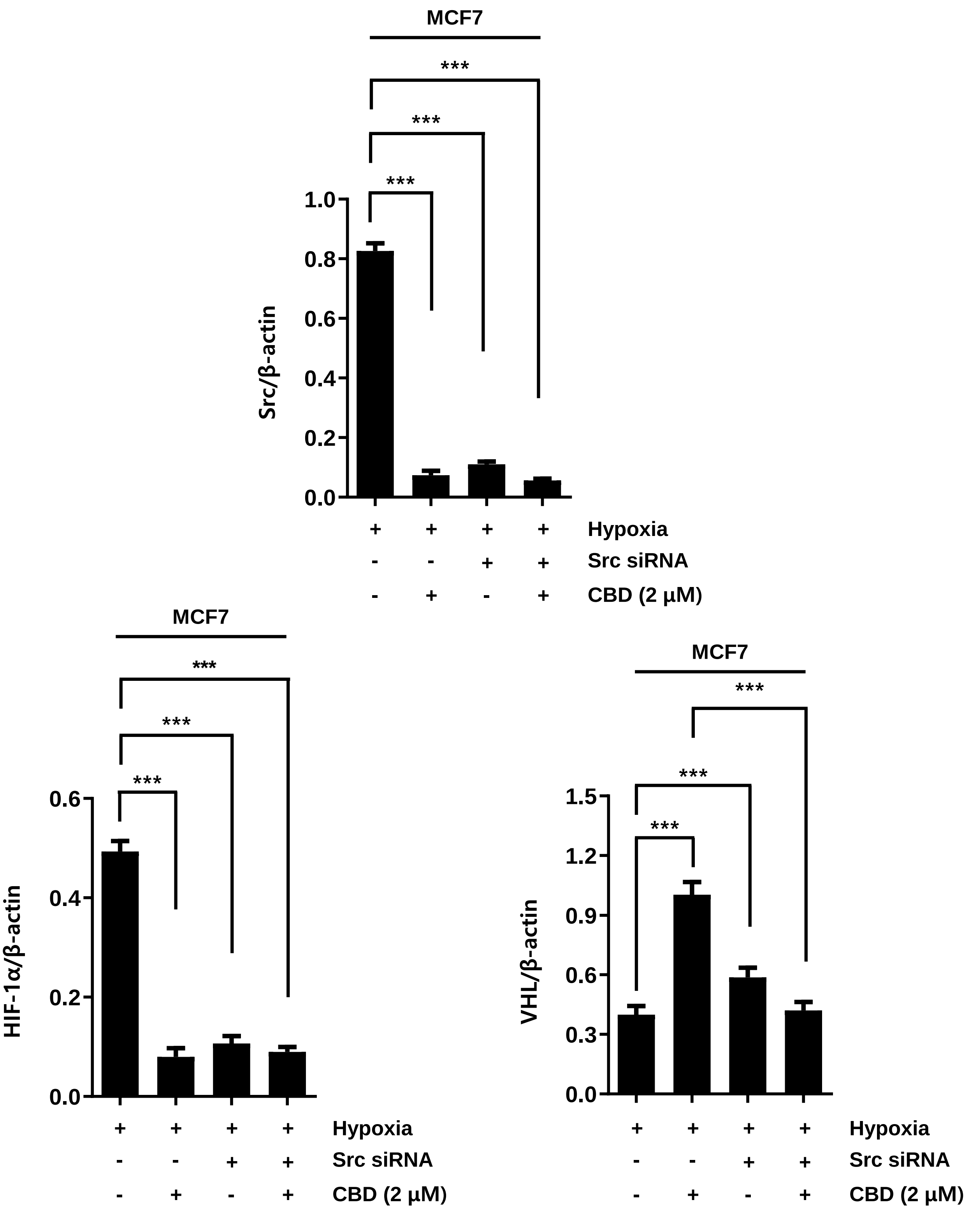

Figure S10. Quantifications and statistics of western blot assays relative to Figure 3D. Blots were quantified using ImageJ software and normalized with β-actin bands as a loading control. Statistical significance was defined as P values < 0.05 (*, **, and *** indicate P < 0.05, P < 0.01, and P < 0.001, respectively)

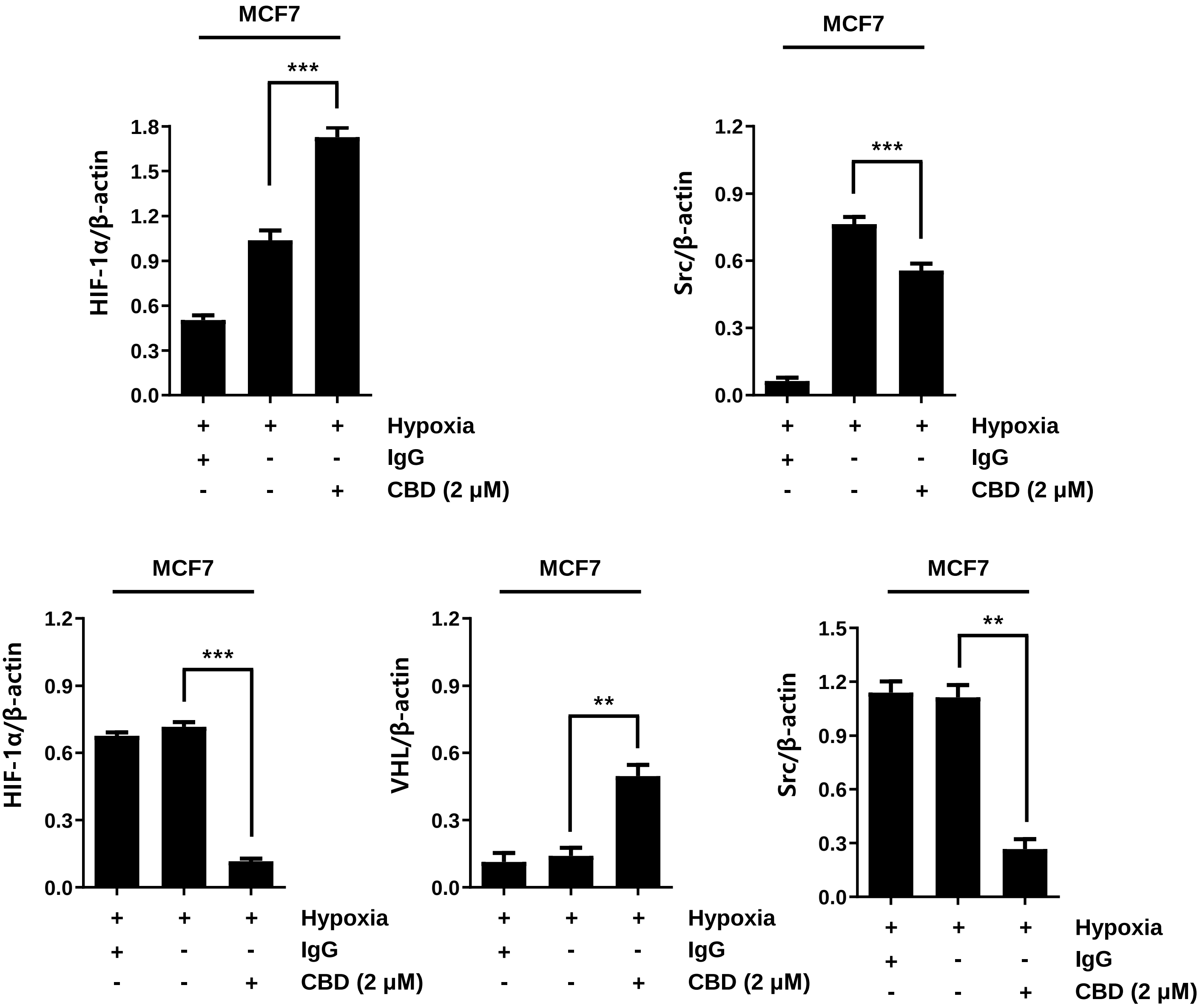

Figure S11. Quantifications and statistics of western blot assays relative to Figure 3E. Blots were quantified using ImageJ software and normalized with β-actin bands as a loading control. Statistical significance was defined as P values < 0.05 (*, **, and *** indicate $P < 0.05$, $P < 0.01$, and $P < 0.001$, respectively)

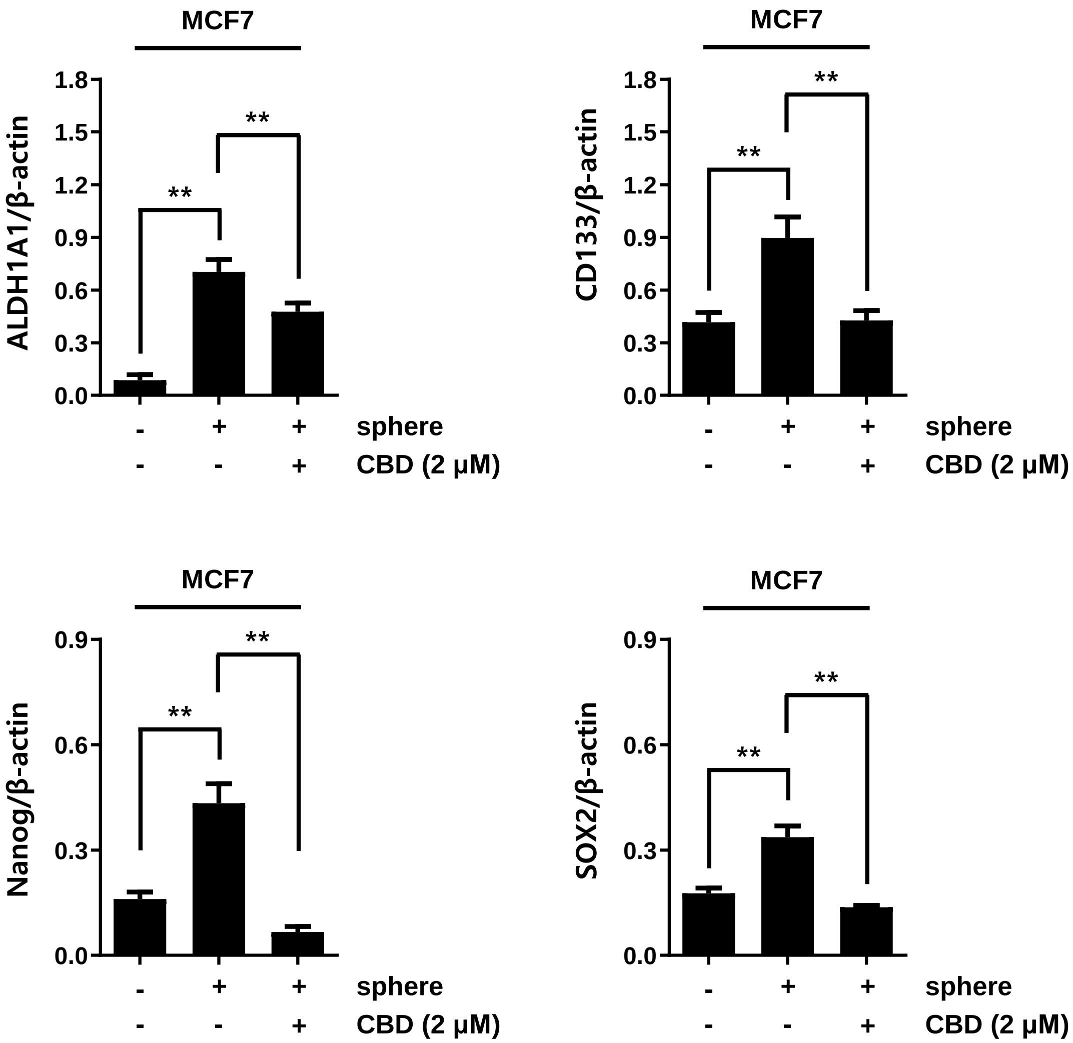

Figure S12. Quantifications and statistics of western blot assays relative to Figure 5F. Blots were quantified using ImageJ software and normalized with β-actin bands as a loading control. Statistical significance was defined as P values < 0.05 (*, **, and *** indicate $P < 0.05$, $P < 0.01$, and $P < 0.001$, respectively)

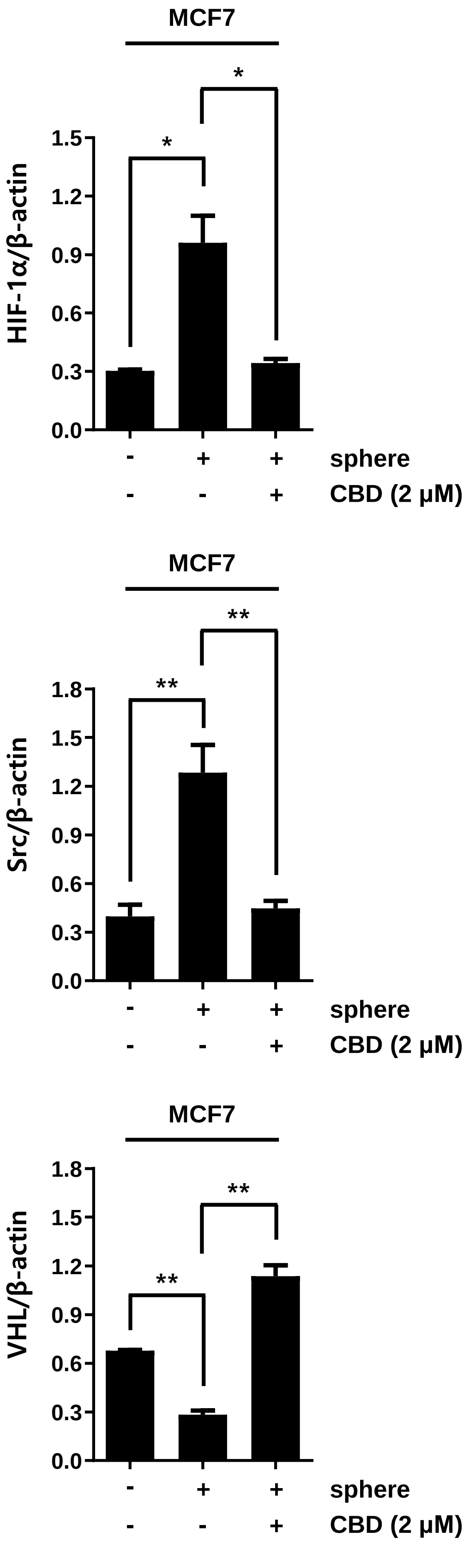

Figure S13. Quantifications and statistics of western blot assays relative to Figure 5G. Blots were quantified using ImageJ software and normalized with β-actin bands as a loading control. Statistical significance was defined as P values < 0.05 (*, **, and *** indicate $P < 0.05$, $P < 0.01$, and $P < 0.001$, respectively)

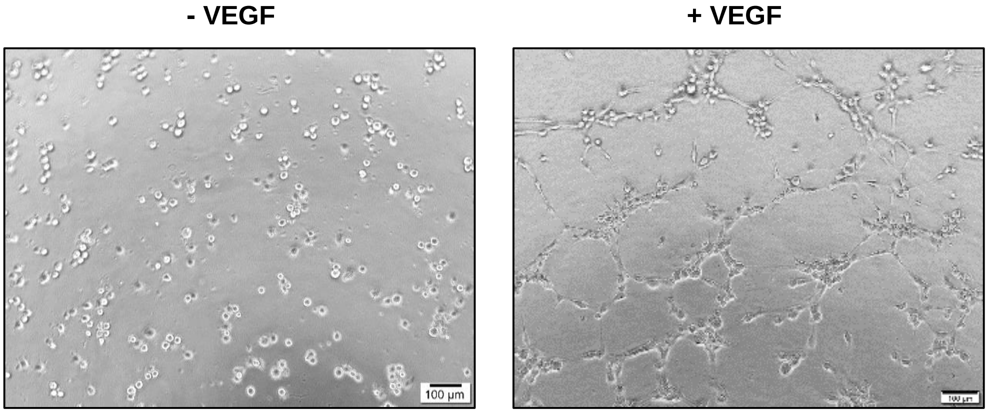

Figure S14. The angiogenic potential was determined by HUVEC tube-formation assay. HUVECs were grown on matrigel-coated 48-well plates with conditioned media with or without VEGF. Capillary tube formation was captured using a light microscope.

Figure 1B

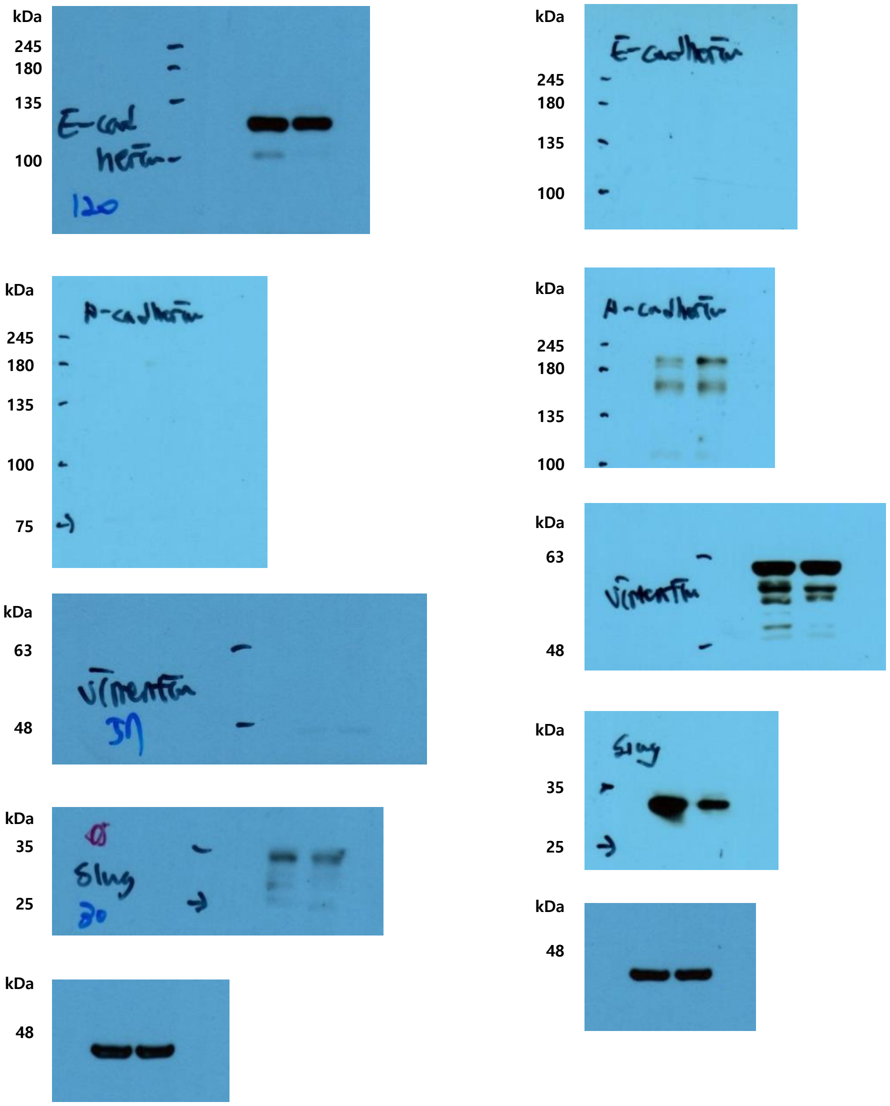
kDa
245
180
135
100
75
63
48
35
25
E-cadherin
N-cadherin
vimentin
Slug

Figure 2A

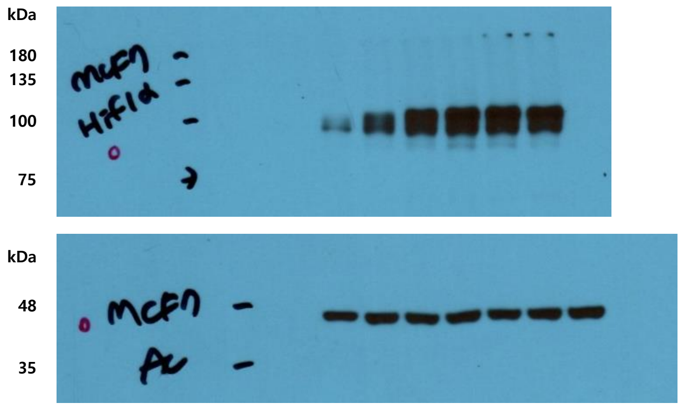

Figure 2B

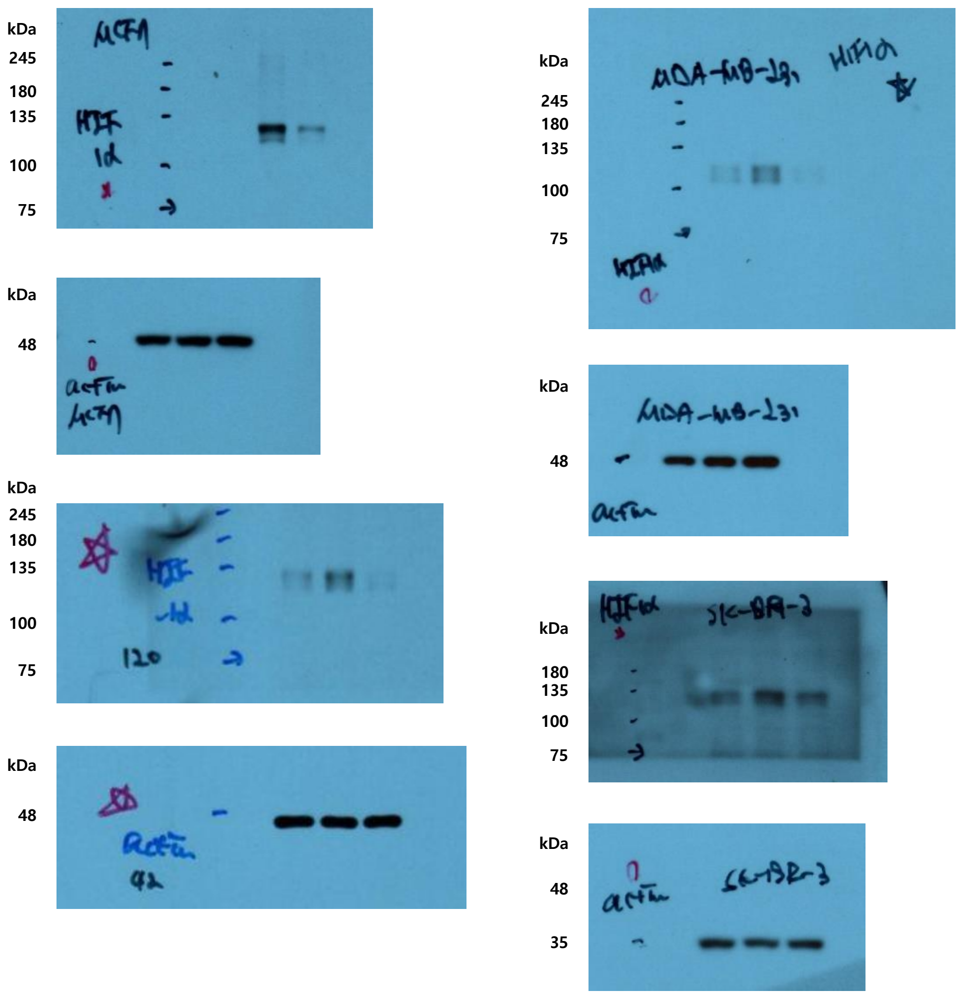

Figure 2C

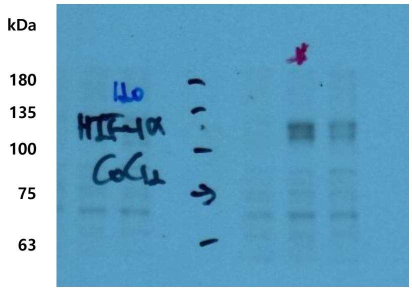

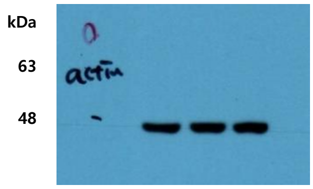

Figure 2D

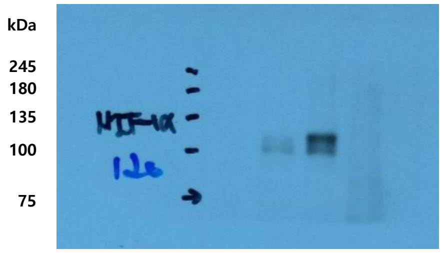

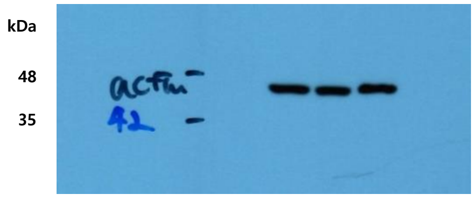

Figure 2G

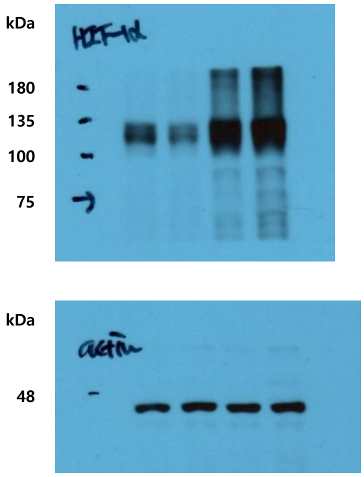

Figure 2H

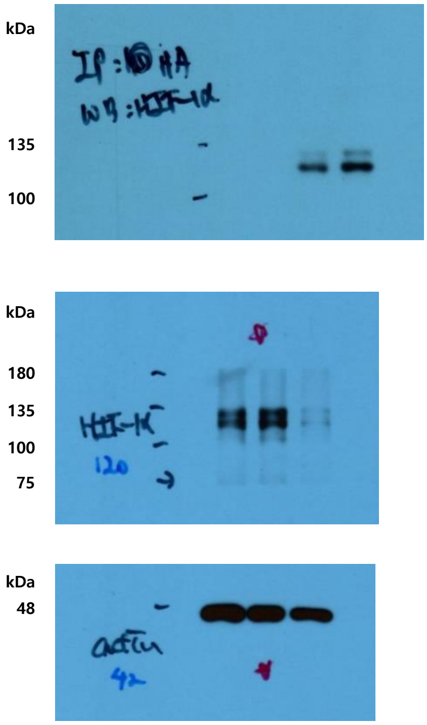

Figure 3A

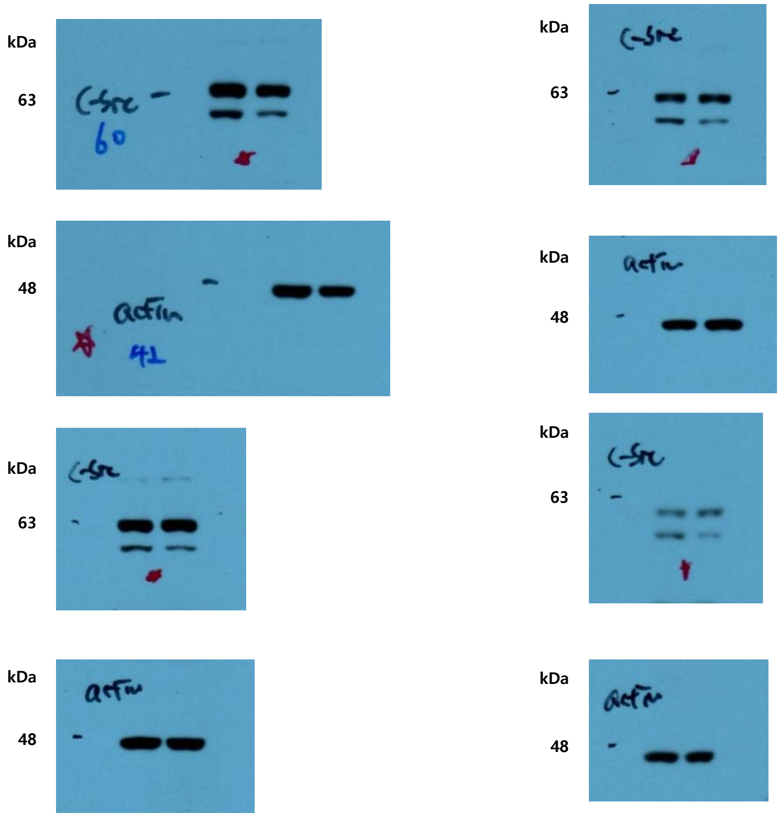

Figure 3C

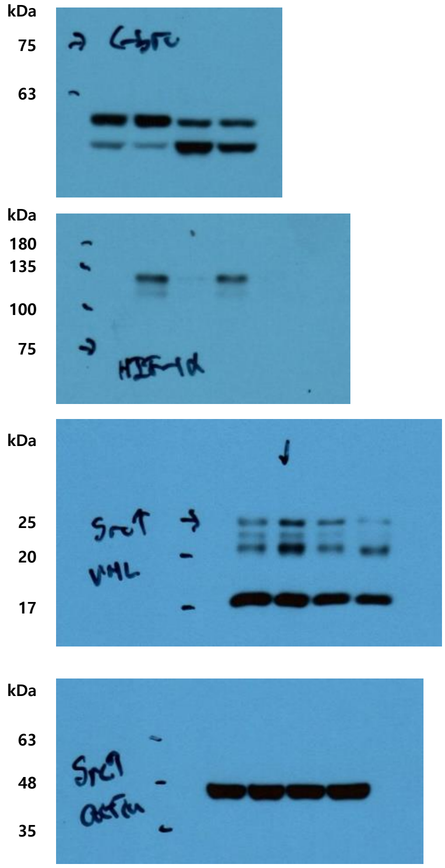

Figure 3D

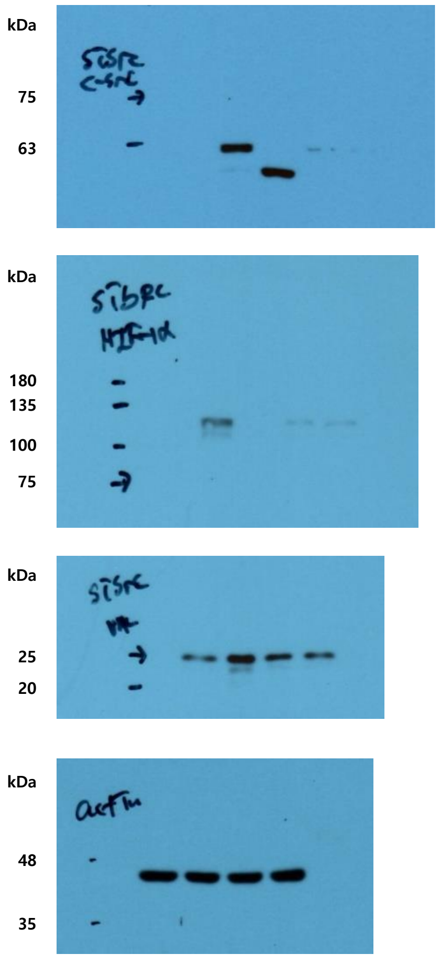

Figure 3E

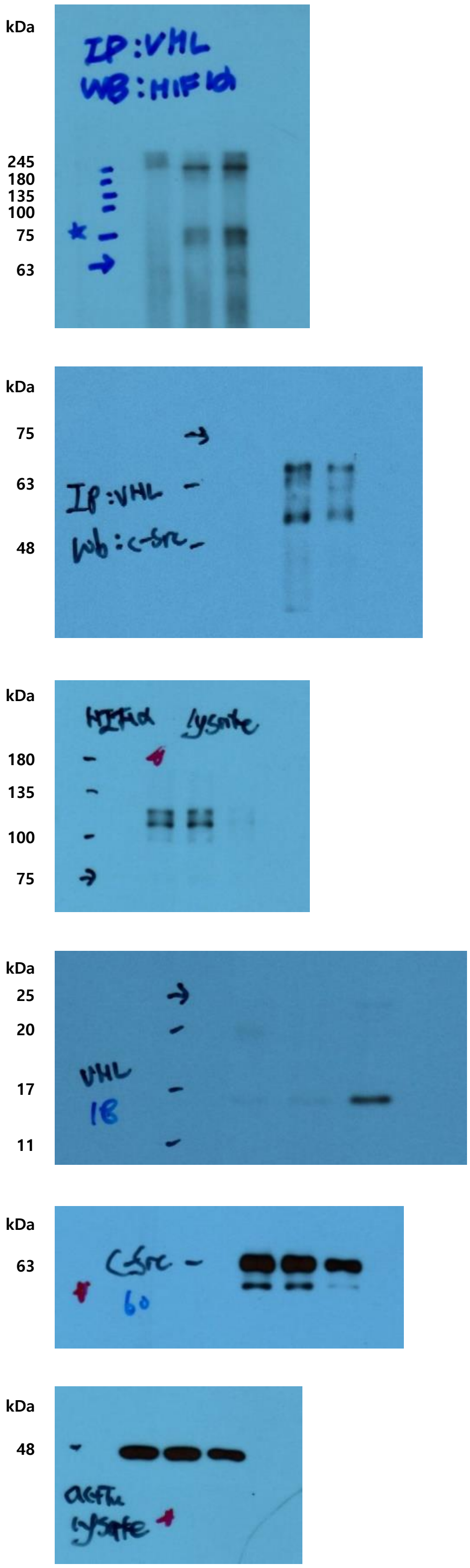
kDa
IP:VHL
245
180
135
100
75
63
kDa
75
63
48
kDa
180
135
100
75
kDa
25
20
VHL
17
11
kDa
63
kDa
48

Figure 5F

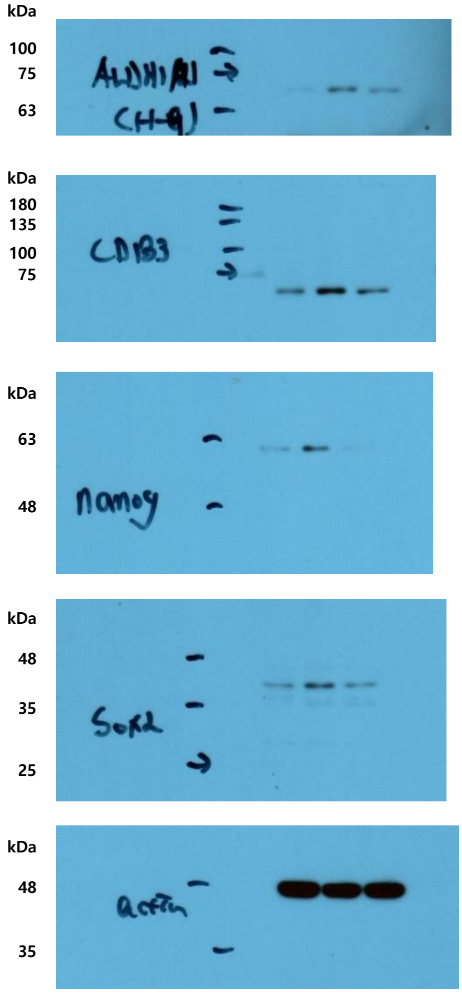

Figure 5G

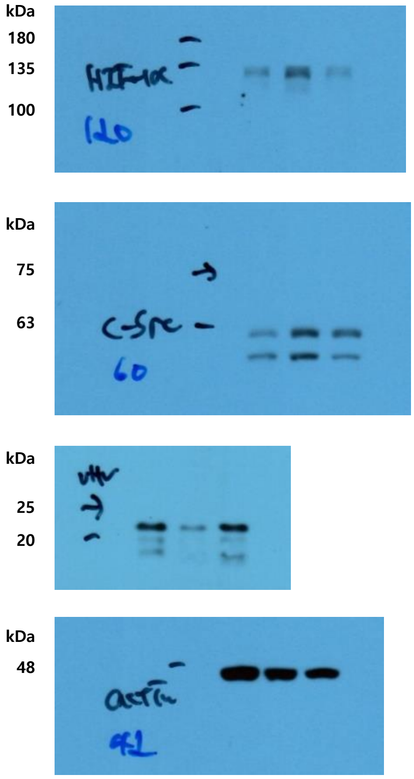

Supplement: Supplementary file 1 [file cancers-13-05667-s001.zip › cancers-1441328-supplementary.pdf]
